# Regulation of the Development and Function of B Cells by ZBTB Transcription Factors

**DOI:** 10.3389/fimmu.2018.00580

**Published:** 2018-03-20

**Authors:** Can Zhu, Ge Chen, Ying Zhao, Xiao-Ming Gao, Jun Wang

**Affiliations:** ^1^Institutes of Biology and Medical Sciences, Soochow University, Suzhou, China; ^2^Department of Pathophysiology, School of Biology and Basic Medical Sciences, Soochow University, Suzhou, China

**Keywords:** B cells, BCL6, ZBTB7A, ZBTB17, ZBTB20, ZBTB32, ZBTB1, ZBTB24

## Abstract

The large ZBTB family comprises a diverse group of transcriptional factors. Several ZBTB proteins have emerged as critical factors that regulate the lineage commitment, differentiation, and function of lymphoid cells as well as many other developmental events. For instance, dysfunctions of ZBTB20 or ZBTB24 have been linked to multisystem failures in humans. Within the B-cell lineage, BCL6, ZBTB7A, ZBTB17, and ZBTB1 regulate the development/differentiation of B cells in both bone marrow and peripheral lymphoid organs, while ZBTB20 and ZBTB32 seem to mainly impact the maintenance of terminal plasma cells. Given the importance of B cells in the prevention and treatment of infectious or autoimmune disorders, we herein summarize the roles of seven ZBTB family members (BCL6, ZBTB7A, ZBTB17, ZBTB20, ZBTB32, ZBTB1, and ZBTB24) in the development, differentiation, and function of B cells as well as the underlying molecular mechanisms.

## Introduction

The development of B cells from hematopoietic stem cells (HSCs) toward memory (Bmem) or plasma cells (PCs) represents a long and highly coordinated, yet flexible, pathway. At multiple steps, prominent transcriptional factors (TFs), including TCF3 (transcription factor 3), EBF1 (early B-cell factor-1), FOXO1 (forkhead box O1), and PAX5 (paired box-5), orchestrate the B-cell fate specification (1, 2). In the bone marrow (BM), “graded” expressions of SPI1 (PU.1) and IKZF1 (IKAROS family zinc finger-1) direct the development of HSCs toward common lymphoid progenitors (CLPs) (3, 4). In CLPs, TCF3 induces the expression of EBF1 and FOXO1 (5–7). EBF1 in turn synergizes with FOXO1 and other TFs such as SPI1 and IRF-4/8 to induce PAX5, which acts at multiple levels to commit cells to B-cell lineage (7–9). Moreover, IKZF1 and FOXO1 regulate IL-7 signaling and the heavy/light-chain gene rearrangements in pro-/pre-B cells (10–12), and mice deficient for IRF-4/8 display a block at the pre-B-to-B transition (13). During these processes, IL-7, provided by mesenchymal stromal cells, promotes the survival and proliferation of early B-cell progenitors (14).

In the periphery, naive B cells are activated with antigen-presenting dendritic cells, proliferate rapidly and form germinal centers (GCs) under the help of follicular helper T (Tfh) cells (15). In GCs, B cells undergo massive proliferation, somatic hypermutation (SHM), and class-switch recombination (CSR) and eventually differentiate into Bmem or PCs, which are finely controlled by appropriate expressions of two sets of TFs with distinct functions (16, 17). The expression of BCL6/BACH2/PAX5 maintains the B-cell phenotype and promotes their proliferation and SHM, whereas IRF4/PRDM1/XBP1 blunts proliferation and facilitates the CSR and differentiation toward PCs or Bmem. The terminally differentiated PCs, guided by CXCR4/CXCL12 chemoattraction, exit from GC and home to BM, where they live for long periods in the presence of IL-6 produced by stromal cells (18).

**Figure 1:**
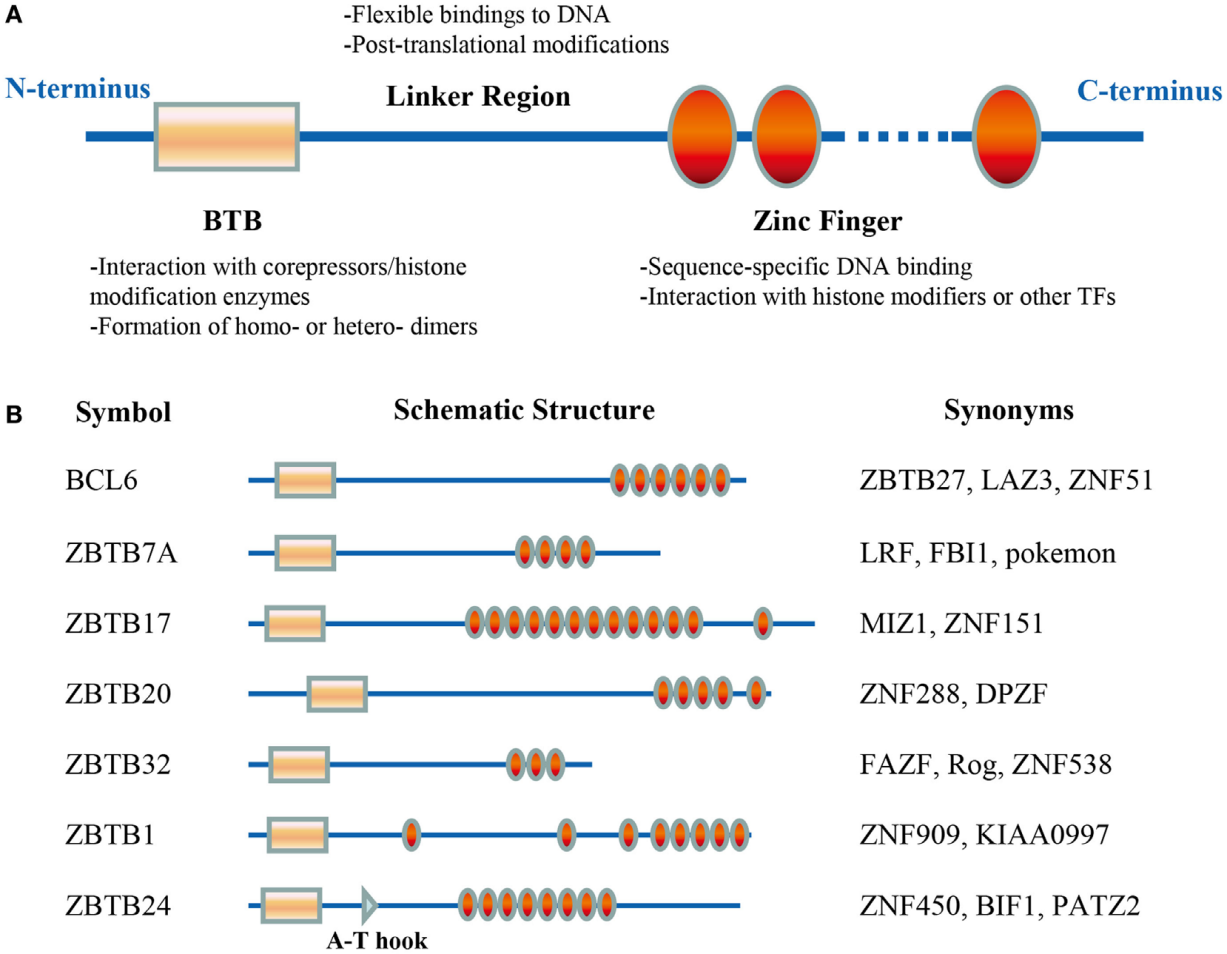
Schematic illustrations of the structure and function of ZBTB protein domains. The BTB domain, located at the N-terminus, mediates the protein–protein interactions. The C-terminal zinc fingers (ZFs) mainly mediate the binding to DNA, but they may interact with histone modifiers or other transcriptional factors (TFs) as well. The linker domain is unstructured and often targeted for posttranslational modifications **(A)**. **(B)** Functional domains of the seven ZBTB proteins discussed in this review. ZBTB24 contains an additional A-T hook domain.

ZBTB proteins, characterized as having one or more C-terminal C_2_H_2_/Krüppel-type zinc finger (ZF) domains and an N-terminal BTB (broad-complex, tram-track, and bric-a-brac) domain (Figure 1A), are an evolutionarily conserved family of TFs. The BTB domain directly interacts with different corepressors and histone/protein modification enzymes, including NCOR-1/2 (nuclear receptor corepressor-1/2), BCOR (BCL6 corepressor), CTBP1, SIN3A, HDAC, and CUL3, and thus mediates chromatin remodeling and gene silencing/activation (19, 20). This domain also mediates the formation of homo- or heterodimers among ZBTB members, which is critical for their protein stability, cellular localization, and transcriptional activity (21). By contrast, the ZF domains determine the transcriptional specificity of ZBTB proteins through binding to regulatory regions in targeted genes in a sequence-specific manner (22). Notably, the DNA-binding sequence may vary among different cell types/subsets. Genome-wide analysis has revealed that BCL6 binds to different binding motifs in primary Tfh, Th9, macrophages, and B cells (23). Additionally, ZFs may bind to histone modifiers or other TFs as well. For example, the interaction between ZFs and MYC is required for the gene repressing activity of ZBTB17 (24). Furthermore, the poorly conserved linker region, between the BTB and ZF domains, is often targeted for posttranslational modifications and regulates the stability and flexibility of ZBTB–DNA complex (22).

## ZBTB Proteins Regulate the Development, Differentiation, and Function of B Cells

Approximately 60 ZBTB proteins have been identified, many of which have been implicated in diverse cellular processes including DNA damage responses and cell cycle progression, and in a variety of developmental events such as gastrulation and limb formation (25–27). Moreover, since the discovery of chromosomal translocations of BCL6 in lymphoma (28), critical roles of at least 12 *Zbtb* genes in hematopoietic lineage determination and differentiation have been disclosed (22). In the context of lymphoid cells, ZBTB7B (ThPOK) and ZBTB16 (PLZF) mainly regulate the lineage commitment and function of T or innate lymphoid cells (29–31), while ZBTB20 appears to only impact the function of B cells (32, 33). This review will focus on current findings on seven ZBTB proteins with reported roles in B-cell development and function: BCL6 (ZBTB27), ZBTB7A, ZBTB17, ZBTB20, ZBTB32, ZBTB1, and ZBTB24 (Figure 1B).

### BCL6

BCL6 (B cell lymphoma-6, also known as ZBTB27), was first identified as an oncogene frequently translocated/hypermutated in diffuse large B cell lymphoma (DLBCL) and follicular lymphoma (FL) cells (28, 34–36). The transformative activities of BCL6 are largely attributed to its transcriptional repression of genes involved in DNA damage responses and cell cycle progressions (37).

**Figure 2:**
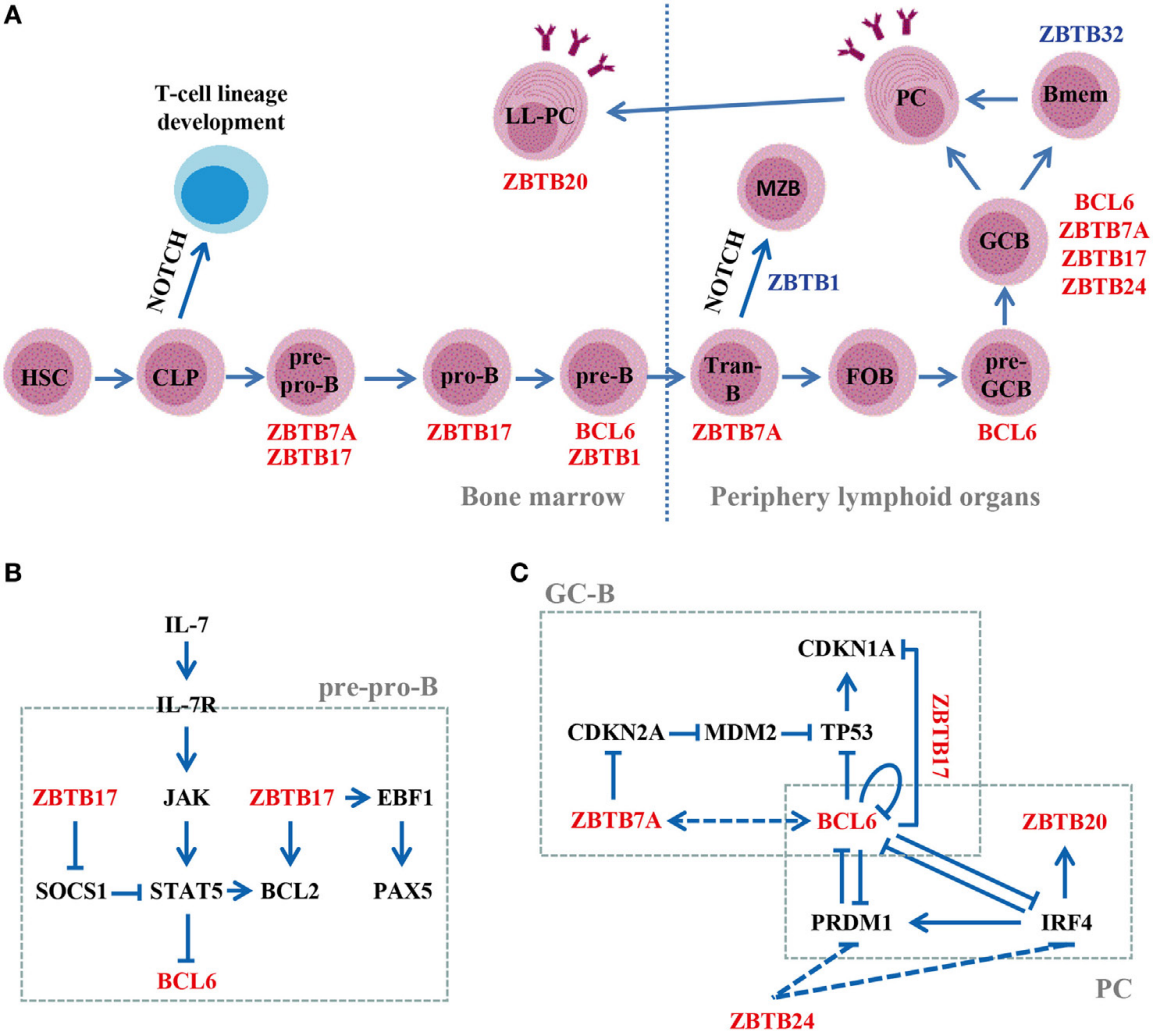
Roles of ZBTB proteins in B-cell development, differentiation, and function. **(A)** A schematic view of the stages most affected by ZBTB proteins along the B-cell development program. ZBTB7A, ZBTB17, ZBTB1, and BCL6 regulate early B-cell development in the bone marrow, while ZBTB7A, BCL6, ZBTB17, ZBTB1, ZBTB20, ZBTB24, and ZBTB32 function in mature B-cell compartments. Positive/negative regulators are indicated in red/blue, respectively. **(B)** Regulation of the IL-7R signaling pathway by ZBTB17 in pre-pro-B cells. ZBTB17 plays a dual role by inducing BCL2 while repressing SOCS-1. **(C)** Transcriptional regulation of genes important for the GC-B or plasma cell (PC) development. Dotted lines represent data obtained in cell lines, and the *in vivo* functional relevance remains to be confirmed. Tran-B, transitional B cells; LL-PC, long-lived PC.

Beyond driving the development of Tfh cells, the B-cell-intrinsic role of BCL6 in GC reactions is highlighted by the impaired GC formation and significantly reduced GC-B cells in mice with a specific deletion of BCL6 in B (mb1-Cre) or GC-B (Cγ1-Cre) cells after immunization with T-cell-dependent (TD) antigens (38). BCL6 directly binds to and represses the transcription of key trafficking receptors S1PR1 and GPR183 by recruiting HDAC2 through the RD2 domain (amino acids 350–395), and thereby governs the commitment of activated B cells to form GCs (Figure 2A) (39). Once GCs are established, BCL6 promotes the proliferation, SHM, and CSR of GC-B cells by inhibiting DNA damage sensing and cell cycle/apoptosis checkpoint genes, including TP53, CHEK1, ATR, and CDKN1A (Figure 2C) (37). Notably, BCL6 exerts these functions in GC-B cells through BTB-dependent recruitment of NCOR-1/2 and BCOR corepressors (40–42). Moreover, BCL6 prevents the premature activation of proliferating GC-B cells in the dark zone by repressing CD69, STAT1, and CD80 (43). BCL6 also maintains the phenotype of GC-B cells by directly silencing the expression of TFs essential for PC differentiation, such as PRDM1 and IRF4 (Figure 2C) (44, 45). Additionally, BCL6 cooperates with BACH2 to regulate GC responses. The BCL6–BACH2 complex not only maintains BACH2 protein stability, but also promotes each other’s binding to regulatory regions of *PRDM1* in GC-B cells (46).

BCL6 is also required for pre-B cell self-renewal and the formation of a diverse B-cell repertoire in BM (Figure 2A). Upon productive V_H_-DJ_H_ rearrangement, signaling through pre-BCR upregulates BCL6, which protects pre-B cells from DNA damage-induced apoptosis during Ig gene recombination by repressing CDKN2A and TP53. In the absence of BCL6, the pool of new BM immature B cells is significantly reduced in size and clonal diversity (47).

It has been shown that IRF8 and SPI1 may contribute to BCL6 induction in antigen-engaged pre-GC-B cells, while IRF4 and PRDM1 repress BCL6 in light zone GC-B cells (16, 48). Moreover, the binding of BCL6 to its own 5′ regulatory region forms an autoregulatory circuit that limits its own expression in GC-B cells (Figure 2C) (49). In addition to these transcriptional regulations, posttranslational modifications of BCL6, such as phosphorylation or acetylation that eventually leads to protein degradation or impaired ability to recruit corepressors, are important in fine-tuning its expression and function as well (16). Together, these multiple layers of regulation not only represent safe-keeper mechanisms in maintaining proper genome integrities of GC-B cells undergoing SHM and CSR, but also ensure their terminal differentiation toward Bmem or PCs.

### ZBTB7A

ZBTB7A, also known as leukemia/lymphoma-related factor (LRF), is broadly expressed and acts as a master regulator of cellular differentiation and lineage fate decisions in hematopoiesis (22). Mice with an inducible deletion of ZBTB7A exhibit an augmented development of extrathymic CD4^+^CD8^+^ double-positive (DP)-T cells in the BM at the expense of B-cell lymphopoiesis, which is reversed by blocking NOTCH signaling (50). ZBTB7A-deficient B220^+^ pre-pro-B cells express significantly reduced levels of pre-BCR components (Igα/β and VpreB1) and RAG recombinases, and are unable to progress further into pro-B cells. By contrast, they resemble normal thymic CD4^−^CD8^−^ double-negative (DN) T cells and readily differentiate into DP-T cells when cocultured with NOTCH ligand DLL1-expressing stromal cells (50). Moreover, mice with a specific ablation of ZBTB7A in B cells after the pro-B stage (Mb1-cre) display grossly normal BM B-cell development, but exhibit a skewed differentiation toward marginal zone (MZB) at the expense of follicular (FOB) B cells in secondary lymphoid organs (51). ZBTB7A-deleted transitional B cells express increased levels of NOTCH target HES1, and consequently, repressing NOTCH2–DLL1 axis largely normalizes the disturbed FOB vs. MZB differentiation *in vivo*. Collectively, these data indicate that ZBTB7A is essential for both early B-cell lineage commitment at progenitor stages in BM and the fate decisions between FOB vs. MZB at the transitional B-cell stage in the periphery (Figure 2A) by antagonizing NOTCH signaling, although it is dispensable for the maintenance of “committed” B-cell precursors in BM. Notably, blocking DLL4, but not DLL1, rescues the aberrant lymphoid differentiation, but not the anemia phenotype, in ZBTB7A-knockout mice (52). Together, these data indicate that ZBTB7A regulates NOTCH signaling in a ligand- and/or cell type-dependent fashion.

Like BCL6, ZBTB7A is highly expressed in normal GC-B and malignant DLBCL/FL cells, and regulates GC responses as well (Figure 2A) (53, 54). B-cell-specific deletion of ZBTB7A (mb1-cre) results in significantly reduced number of GC-B cells in mice upon immunization with TD antigens. However, the overall GC structures, albeit small, remain intact and a few GC-B cells are still observed in these mice (51), indicating that ZBTB7A is required for the maintenance and function of GC-B cells rather than their development *per se*. Accordingly, ZBTB7A-deficient GC-B cells exhibit reduced proliferation and increased apoptosis *in vivo* as ZBTB7A suppresses the TP53 signaling in these cells under genotoxic stress (i.e., SHM and CSR) by repressing CDKN2A (Figure 2C). Notably, ZBTB7A forms an obligate dimer in B cells, which is essential for its function. The high protein yet low mRNA levels of ZBTB7A in GC-B cells indicate that its expression is mainly controlled *via* posttranslational mechanisms (51).

### ZBTB17

ZBTB17, commonly known as MYC-interacting zinc finger protein-1 (MIZ1), is indispensable for early embryonic development during gastrulation (55). Mice with a transcriptionally inactive form of ZBTB17 (Vav-cre mediated deletion of the BTB domain) in hematopoietic cells, display a severely defected lymphoid, but not myeloid development (56, 57). ZBTB17-deficient lymphoid progenitors are arrested at the stage of pre-pro-B or DN-T cells, mainly because they fail to activate JAK–STAT5 pathway and do not upregulate the anti-apoptotic gene BCL2 upon IL-7 stimulation. ZBTB17 represses the STAT5 inhibitor SOCS1 (suppressor of cytokine signaling-1) while upregulates BCL2 by directly binding to their promoters, and thus regulates the IL-7R signaling pathway (Figure 2B), which is critical for the development of CLPs toward T or B-cell lineages (56, 57). Moreover, ZBTB17-deficient CLPs express low levels of genes essential for the early commitment and differentiation of B cells, including TCF3, EBF1, PAX5, and RAG-1/2. Consequently, only the combined reexpression of both BCL2 and EBF1 drives ZBTB17-deficient precursors to develop further into CD19^+^ B cells (57). Because mice deficient for ZBTB17 from the stage of pro-B cells (CD19-cre) have normal B cell development (57), ZBTB17 seems to precisely promote the pre-pro-B to pro-B transition by regulating IL-7R/JAK/STAT5/BCL2 signaling (Figure 2A). This pathway not only regulates cell survival, but also induces the expression of EBF1/PAX5/RAG axis that drives the differentiation along the B-cell lineage (Figure 2B). Moreover, a recent study indicated that ZBTB17 may promote the survival of pre-T and pro-B cells undergoing V(D)J recombination by directly activating ribosomal protein L22, which binds to TP53 mRNA and negatively regulates its translation (58). In addition, ZBTB17 represses CDKN1A and cell cycle arrest in GC-B cells in collaboration with BCL6 (Figure 2C) (59).

### ZBTB20

ZBTB20 is a multifunctional protein as ZBTB20-deficient mice exhibit hypoglycemia, growth retardation, and premature lethality (60, 61). Within the B-cell lineage, ZBTB20 is specifically expressed in peritoneal B1 and GC-B cells and peaks in long-lived BM PCs (32). IRF4 drives the expression of ZBTB20 in B1 and PCs by directly binding to its promoter (Figure 2C), but how ZBTB20 is induced in GC-B cells remains unknown (32). ZBTB20 is dispensable for B-cell development, the initial GC responses and the generation of affinity-matured PCs. However, ZBTB20 deficiency results in a profound defect in long-term antibody responses, which is mainly attributed to the decreased survival of BM PCs (Figure 2A) (32, 33). Reduced levels of MCL1, a potent mediator of PC survival, was observed in singly sorted ZBTB20-deficient PCs (33), but not at the bulk cell population (32). Interestingly, the impact of ZBTB20 on the maintenance of PCs is adjuvant (alum)-dependent, because immunization of mice with TLR ligand-based adjuvant abrogates these effects. This may, in part, be due to the upregulation of CD37, which overcomes the survival defect in ZBTB20-deficient PCs (33).

Recently, heterozygous missense mutations in *ZBTB20*, all located in the DNA-binding ZF domain, were discovered to be responsible for primrose syndrome with multisystem failures (62). Monitoring B-cell populations and antibody titers after vaccination in these patients would shed light on its role in human B-cell development and function.

### ZBTB32

*ZBTB32* has emerged as the most consistent and highly expressed gene in human activated B-cell type of DLBCL (63), possibly due to its ability to facilitate the immune-evasion of these cells by transcriptionally repressing *CIITA* (MHC-II transactivator) (64). ZBTB32 is rapidly induced and cooperates with PRDM1 in silencing *CIITA* by directly binding to its promoter region during the differentiation of PCs *ex vivo* (64). However, primary antibody responses are intact in ZBTB32-knockout mice after immunizations (64, 65), indicating that PRDM1 may compensate for ZBTB32 loss in PC differentiation *in vivo*. Nonetheless, ZBTB32-deficient Bmem-mediated recall responses occur more rapidly and persist longer than do control responses, which may be attributed to their superior ability to present antigen and receive T-cell help as activated ZBTB32-deficient Bmem express high levels of genes involved in lysosomal function/antigen-processing (*Cts-a/b/d*) (65). Moreover, secondary PCs derived from ZBTB32-depleted Bmem express increased levels of proteins promoting cell cycle progression (E2F3 and PCNA) and mitochondrial function (MRPL-18/22/51), and thereby exhibit a cell-intrinsic survival advantage relative to control counterparts (65). ZBTB32 thus is not required for primary antibody responses, but it restricts the duration of antibody recall responses (Figure 2A). Given the absence of ZBTB32 expression in PCs (65), ZBTB32 appears to shorten their lifespan during the upstream activation phase.

### ZBTB1

Three studies recently showed that ZBTB1 deficiency results in a severely impaired lymphoid development, and to a less extent, myeloid development in mice (66–68). The expression of ZBTB1 in B-cell subsets is strikingly increased from the pro-B to pre-B stage, and subsequently declines upon further differentiation (67). ZBTB1 deficiency leads to a significantly reduced number of pre-B cells (Figure 2A), which can be reversed by the concomitant overexpression of BCL2 or knockout of TP53 (66–68). ZBTB1 enhances CHEK1 phosphorylation in replicating murine B cells, which blocks DNA synthesis to allow for lesion repair, and thereby prevents the accumulation of DNA damage and TP53-mediated apoptosis (68). In addition, ZBTB1-deficient mice exhibit an increase of MZB that can not be reversed by BCL2 overexpression (Figure 2A) (67, 68), indicating that ZBTB1 regulates FOB vs. MZB differentiation in the periphery *via* different mechanisms.

In human UV-irradiated Hela cells, no effect of ZBTB1 on CHEK1 phosphorylation is observed, whereas it interacts with TRIM28/NuRD complex and guides them to damage sites, leading to chromatin remodeling, increased RAD18 accessibility and initiation of translesion DNA synthesis (69). It is likely that this mechanism is also employed by ZBTB1 to maintain the genome integrity of primary B cells during replication and differentiation.

### ZBTB24

*ZBTB24* has been recently identified as the causative gene in patients with immunodeficiency, centromeric instability, and facial anomalies syndrome type 2 (ICF2), a rare autosomal recessive disease (70). Most ICF2 patients harbor *ZBTB24* nonsense mutations that generate a premature stop codon, and suffer from recurrent respiratory and gastrointestinal infections due to hypogammaglobulinemia (70–73). Despite the normal amount of circulating T/B cells, detailed immunological phenotyping has revealed a lack of GC structure and circulating CD19^+^CD27^+^ Bmem in ICF2 patients (70–73). ZBTB24 is highly expressed in the human B-cell compartment (70). Moreover, its downregulation hampers the cell cycle progression in human GC-derived B lymphoma Raji cells *via* upregulating the expression of IRF4 and PRDM1 (Figure 2C). Notably, ZBTB24 functions independent of BCL6 as it neither heterodimerizes with nor affects the expression/transcriptional activity of BCL6 (74). Given the lack of GC in ICF2 and the critical roles of IRF4/PRDM1 in GC responses (70–73), ZBTB24 appears to control the *in vivo* Bmem development *via* regulating the proliferation and/or terminal differentiation of human GC-B cells (Figure 2A). Given the high expressions of ZBTB24 in BM pre-B and immature B cells (70), and the significantly increased number of circulating immature B cells in ICF patients (75), ZBTB24 may regulate early B-cell development as well. Because the conventional knockout of *zbtb24* is an embryonic lethal mutation (76), mice with an inducible or cell/stage-specific deletion of *zbtb24* would be useful in addressing its role in B cells *in vivo*.

## Concluding Remarks

Herein, we have discussed recent findings on seven ZBTB family proteins in the development and function of B cells. New studies are required to further decipher the molecular mechanisms responsible for the cell- or stage-specific effect of ZBTB proteins in regulating gene expressions. Elucidating (i) how the expression of ZBTB TFs is transcriptionally regulated, (ii) how ZBTB proteins are posttranslationally modified (i.e., phosphorylated, acetylated, or ubiquitinated), and (iii) how these modifications regulate the stability/activity of ZBTB proteins, will be central to understand their mechanisms of action along the B-cell differentiation program. Moreover, given the overlapped expression and function of BCL6 and ZBTB7A in GC-B cells (54), and the formation of BCL6/ZBTB7A heterodimers in malignant DLBCL/FL cells (53, 77), it is likely that they collaborate in modulating cellular functions (Figure 2C). In addition to transcriptional activity, more attention should be paid to other functions of ZBTB proteins, because they may also directly participate in processes including DNA repair (as reported for ZBTB1) (69). The combination of structural and computational studies will provide new insights into the assembly of ZBTB-containing protein complex and their mechanics of transcriptional-dependent and -independent activities. Finally, inducible and cell/stage-specific manipulations of ZBTB expression/function would help clarify its significance *in vivo*.

Although ZBTB is a conserved family of TFs, we should still be cautious in extrapolating findings obtained on murine model into human settings. For instance, ZBTB24 is dispensable for human embryonic development, whereas its deficiency results in embryonic lethality in mice (76). Nevertheless, detailed phenotypic, functional, and molecular characterizations of B cells from patients with dysfunctional ZBTB proteins, in combination with *in vitro* knockdown or overexpression approaches, will provide invaluable insight into fundamental pathways regulated by ZBTB members in health and disease.

## Author Contributions

CZ and GC collected literatures and drafted the manuscript; YZ discussed the literatures and revised the manuscript; X-MG and JW designed the work, collected literatures, and drafted and revised the manuscript.

## Conflict of Interest Statement

The authors declare that the research was conducted in the absence of any commercial or financial relationships that could be construed as a potential conflict of interest.

## Abbreviations

HSCs, hematopoietic stem cells; Bmem, memory B cells; PCs, plasma cells; TFs, transcriptional factors; BM, bone marrow; CLPs, common lymphoid progenitors; GC, germinal center; SHM, somatic hypermutation; CSR, class-switch recombination; ZF, zinc finger; DLBCL, diffuse large B cell lymphoma; FL, follicular lymphoma; TD, T-cell-dependent; MZB, marginal zone B cells; FOB, follicular B cells; DP, CD4^+^CD8^+^ double-positive; DN, CD4^−^CD8^−^ double-negative; ICF2, immunodeficiency, centromeric instability, and facial anomalies syndrome type 2.
